# Assessment of synaptic loss in mouse models of β-amyloid and tau pathology using [^18^F]UCB-H PET imaging

**DOI:** 10.1016/j.nicl.2023.103484

**Published:** 2023-07-26

**Authors:** Letizia Vogler, Anna Ballweg, Bernd Bohr, Nils Briel, Karin Wind, Melissa Antons, Lea H. Kunze, Johannes Gnörich, Simon Lindner, Franz-Josef Gildehaus, Karlheinz Baumann, Peter Bartenstein, Guido Boening, Sibylle I. Ziegler, Johannes Levin, Andreas Zwergal, Günter U. Höglinger, Jochen Herms, Matthias Brendel

**Affiliations:** a Department of Nuclear Medicine, University Hospital of Munich, Ludwig-Maximilians-University (LMU) Munich, Munich, Germany; b German Center for Neurodegenerative Diseases (DZNE), Munich, Germany; c Munich Cluster for Systems Neurology (SyNergy), Munich, Germany; d Center for Neuropathology, LMU Munich, Munich, Germany; e Roche Pharma Research and Early Development, Neuroscience Discovery, Roche Innovation Center Basel, F. Hoffmann-La Roche Ltd., Basel, Switzerland; f Department of Neurology, University Hospital of Munich, LMU Munich, Munich, Germany; g German Center for Vertigo and Balance Disorders (DSGZ), University Hospital of Munich, LMU Munich, Munich, Germany

**Keywords:** SV2A, PET, Synaptic loss, Tau, Aβ

## Abstract

**Objective:**

In preclinical research, the use of [^18^F]Fluorodesoxyglucose (FDG) as a biomarker for neurodegeneration may induce bias due to enhanced glucose uptake by immune cells. In this study, we sought to investigate synaptic vesicle glycoprotein 2A (SV2A) PET with [^18^F]UCB-H as an alternative preclinical biomarker for neurodegenerative processes in two mouse models representing the pathological hallmarks of Alzheimer’s disease (AD).

**Methods:**

A total of 29 PS2APP, 20 P301S and 12 wild-type mice aged 4.4 to 19.8 months received a dynamic [^18^F]UCB-H SV2A-PET scan (14.7 ± 1.5 MBq) 0–60 min post injection. Quantification of tracer uptake in cortical, cerebellar and brainstem target regions was implemented by calculating relative volumes of distribution (V_T_) from an image-derived-input-function (IDIF). [^18^F]UCB-H binding was compared across all target regions between transgenic and wild-type mice. Additional static scans were performed in a subset of mice to compare [^18^F]FDG and [^18^F]GE180 (18 kDa translocator protein tracer as a surrogate for microglial activation) standardized uptake values (SUV) with [^18^F]UCB-H binding at different ages. Following the final scan, a subset of mouse brains was immunohistochemically stained with synaptic markers for gold standard validation of the PET results.

**Results:**

[^18^F]UCB-H binding in all target regions was significantly reduced in 8-months old P301S transgenic mice when compared to wild-type controls (temporal lobe: p = 0.014; cerebellum: p = 0.0018; brainstem: p = 0.0014). Significantly lower SV2A tracer uptake was also observed in 13-months (temporal lobe: p = 0.0080; cerebellum: p = 0.006) and 19-months old (temporal lobe: p = 0.0042; cerebellum: p = 0.011) PS2APP transgenic versus wild-type mice, whereas the brainstem revealed no significantly altered [^18^F]UCB-H binding. Immunohistochemical analyses of post-mortem mouse brain tissue confirmed the SV2A PET findings. Correlational analyses of [^18^F]UCB-H and [^18^F]FDG using Pearson’s correlation coefficient revealed a significant negative association in the PS2APP mouse model (R = -0.26, p = 0.018). Exploratory analyses further stressed microglial activation as a potential reason for this inverse relationship, since [^18^F]FDG and [^18^F]GE180 quantification were positively correlated in this cohort (R = 0.36, p = 0.0076).

**Conclusion:**

[^18^F]UCB-H reliably depicts progressive synaptic loss in PS2APP and P301S transgenic mice, potentially qualifying as a more reliable alternative to [^18^F]FDG as a biomarker for assessment of neurodegeneration in preclinical research.

## Highlights

• [^18^F]UCB-H was evaluated as a preclinical biomarker of synapse density in Alzheimer’s disease (AD) models.
• Decreased PET signals reflected synapse loss in transgenic mice with tau and Aβ pathology.
• Multitracer comparison showed that [^18^F]FDG but not [^18^F]UCB-H PET is affected by glial metabolism.
• Synaptic density may be a more reliable surrogate of neurodegeneration than glucose metabolism in AD models.

## 1 Introduction

Within the spectrum of neurodegenerative diseases, Alzheimer’s disease (AD) is not only the most prevalent but also amongst the most complex in terms of its intricate pathophysiology. With many dots yet to be connected, current consensus defines plaque and neurofibrillary tangle formation – due to amyloid-beta accumulation and associated with tau hyperphosphorylation, respectively – as two core characteristics of the disease’s pathogenesis (Jack et al., 2018). In a cascade-like process, the formation of amyloid-β deposits presumably represents the central pathology during initial stages, while the aggregation of characteristic tau fibrils occurs at later times (Jack et al., 2010, Lemere and Masliah, 2010). Furthermore, a wealth of studies suggests that inflammatory cells such as activated microglia and reactive astrocytes are also a key element of the neurodegenerative trajectories, exerting both beneficial and harmful effects in a dynamic process over time. Microglia, in particular, likely engage in the clearance of amyloid debris early on but become dystrophic when chronically activated, resulting in wide spread synaptic loss, neuronal apoptosis and propagation of tau aggregates (Kitazawa et al., 2004, Leng and Edison, 2021). Taken together, this simplified pathological model builds the basis of the current A-T-N classification of AD, which aims to integrate three biomarkers (amyloidosis, tau, neurodegeneration) into the diagnostic research protocol, facilitating a uniform quantification independent of clinical symptoms (Jack et al., 2018).

Translated into practice, the challenge of both quantifying and locating these biomarkers in-vivo can be overcome by using target-specific radiotracers in positron emission tomography (PET). This has been done both preclinically as well as in humans, e.g. with [^18^F]flutemetamol to aim at amyloid plaques or [^18^F]AV1451 to locate neurofibrillary tangles (Villemagne et al., 2018). With regards to PET targets representing neurodegeneration, studies in humans have so far taken advantage of the fact that degenerative processes are accompanied by reduced regional glucose metabolism, which is reflected by lower [^18^F]Fluorodesoxyglucose (FDG) uptake (Jack et al., 2018). In preclinical studies, however, FDG-PET delivered controversial results including hyper- and hypometabolism in AD mouse models (Bouter and Bouter, 2019). In this regard, neuroinflammation in transgenic mouse models was associated with increased glucose metabolism of astroglial immune cells (Choi et al., 2021, Xiang, 2021, Zimmer et al., 2017). This discrepancy between preclinical and human PET imaging results substantiates the need to identify alternative biomarkers for regional assessment of neurodegeneration in preclinical research, which could also translate to more reliable determination of regional neurodegeneration in humans. In the present study, we aimed to investigate the potential use of [^18^F]UCB-H as an alternative neurodegeneration biomarker of AD models. As a modification of the antiepileptic drug levetiracetam, this radiotracer specifically targets the synaptic vesicle glycoprotein 2A (SV2A), which is spread out ubiquitously in synaptic terminals across the brain (Rossi, 2022). Consequently, PET imaging of decreased [^18^F]UCB-H uptake facilitates the in-vivo visualization of synaptic decline in various neurodegenerative diseases. Here, previous preclinical as well as human studies have shown that decreases in [^18^F]UCB-H binding is congruent with synaptic loss in brain regions typically affected in AD (Xiong, 2021, Bastin et al., 2020).

On the basis of this observation, we sought to explore the synaptic loss representing the degenerative pathology in two distinct mouse models: one with amyloid-beta (PS2APP) and one with tau (P301S) pathology. Furthermore, we validated our in-vivo findings histologically by using immunostaining of SV2A in the same mouse cohort. Lastly, we also assessed the relationship between [^18^F]UCB-H and [^18^F]FDG binding in both mouse models and explored a potentially confounding influence by microglial activation in PS2APP transgenic mice.

## 2 Materials and methods

### 2.1 Radiochemistry

[^18^F]UCB-H was synthesized on a Trasis AllinOne (Ans, Belgium) automated synthesis unit (ASU) with 3 single-use disposable manifolds connected in series with a total of 18 valves. The manifolds were clamped into the correct position at the module. The software prompts were followed to run the cassette test. All reagents were assembled on the pre-defined positions of the manifold (position 2: eluent, position 8: precursor solution pre-mixed with TEMPO in MeCN, position 11: MeCN, position 12: saline bag, position 15: EtOH, position 16: sodium ascorbate) followed by HPLC priming and preliminary steps. No carrier added [^18^F]fluoride was produced via ^18^O(p, n)^18^F reaction by proton irradiation of ^18^O-enriched water and delivered to the activity inlet reservoir. The precursor is labelled by replacement of the iodonium aryl triflate leaving group by [^18^F]fluoride in a S_N_Ar reaction. [^18^F]UCB-H is purified by reversed phase semi-preparative HPLC (Phenomenex Luna C18(2) column, 5 μm, 10 × 250 mm; isocratic elution with 61% (v/v) / 39% (v/v) acetonitrile; flow: 5 ml/min) and trapped on a SPE cartridge. The cartridge is rinsed and [^18^F]UCB-H is eluted with ethanol and saline. The formulated product solution was transferred to a dispenser and filtered through a Merck 0.22 µm Cathivex-GV sterile filter. The product was obtained in radiochemical yields of 32 ± 5.0 % non d.c. (n = 12) within a synthesis time of 55 min. The RCP was > 99%.

### 2.2 Animals

We used a total of 49 male transgenic mice in this study, 29 of which were homozygous B6.PS2APP (PS2APP) mice (Ozmen et al., 2009), while the other 20 belonged to a homozygous P301S mutant strain (Allen et al., 2002). Furthermore, 12 male C57Bl/6 wild-type (WT) mice were used as a control group. More detailed information on each mouse model is provided in the Supplementary Material. All animals were exposed to a 12 h: 12 h light–dark cycle and their housing environment was assured to maintain room temperature and approximately 50% humidity. Food and water were provided in standard pellets with unrestricted access (Ssniff, Soest, Germany).

### 2.3 Small-Animal PET

#### 2.3.1 Study Overview

The preclinical procedure was approved by the local animal care committee of the Government of Upper Bavaria. All experiments were conducted on the basis of the National Guidelines for Animal Protection and constantly supervised by a veterinarian. Small-animal PET scans with [^18^F]UCB-H were carried out dynamically 0–60 min after radiotracer injection via a tail vein (14.7 ± 1.5 MBq). A detailed overview of each groups’ characteristics is given in Table 1. Additional static [^18^F]FDG PET scans (30–60 min p.i.) were performed on a subset of transgenic and wild-type mice. All mice were deprived of food at least two hours before scanning and blood glucose levels were measured prior to each PET scan. These showed no significant difference between P301S (4 m: 150 ± 26 mg/dL; 8 m: 156 ± 17 mg/dL) and PS2APP mice (4 m: 137 ± 15 mg/dL; 7 m: 134 ± 9 mg/dL; 13 m: 137 ± 12 mg/dL; 19 m: 131 ± 11 mg/dL) when compared to wild-type controls (145 ± 16 mg/dL), irrespective of the age. For more details on the scanning procedure, we refer to our previous study (Xiang, 2021). Furthermore, a subset of PS2APP mice (n = 18) received a third scan with [^18^F]GE180 60–90 min after injection. Once the final PET scan was accomplished, all mice were anaesthetized and perfused with phosphate-buffered saline. To conduct immunohistochemical analysis, the brain was preserved using paraformaldehyde 4%.

**Table 1:** Overview of the cross-sectional quantitative SV2A analysis. Small-animal PET V_T_ ratios are shown for [^18^F]UCB-H. Significant differences in PS2APP and P301S mice versus wild-type controls are indicated by *P ≤ 0.05; **P ≤ 0.01; ***P ≤ 0.001.

Mouse Model	Age (mo)	[^18^F]UCB-H Small Animal PET (n)	Cerebellum [^18^F]UCB-H signal (V_Tr CB_)	Brainstem [^18^F]UCB-H signal (V_Tr BS_)	Temporal Lobe [^18^F]UCB-H signal (V_Tr TL_)
PS2APP	4.4	5	0.72 ± 0.05	0.76 ± 0.02	0.88 ± 0.04
	7.1	7	0.69 ± 0.09	0.74 ± 0.05	0.87 ± 0.07
	13.5	10	0.62 ± 0.05***	0.74 ± 0.02	0.79 ± 0.07**
	19.8	7	0.60 ± 0.05***	0.74 ± 0.01	0.77 ± 0.06**

P301S	4.3	6	0.66 ± 0.05*	0.70 ± 0.03**	0.80 ± 0.09
	8.4	14	0.65 ± 0.05**	0.71 ± 0.02**	0.80 ± 0.08*

WT	12.7	12	0.75 ± 0.08	0.75 ± 0.04	0.90 ± 0.10

#### 2.3.2 PET data Acquisition, reconstruction and analysis

For all PET data acquisition, reconstruction, and image pre-processing were conducted according to an established, standardized protocol (Overhoff, 2016). The PET analyses were performed using PMOD (v3.5, PMOD technologies, Basel, Switzerland). As part of the PET data quantification, we first defined the temporal lobe, cerebellum, and brainstem as three target regions of interest. Both the cerebellum and brainstem volume-of-interest (VOI) were drawn from the standardized Mirrione atlas (Ma et al., 2005). A predefined VOI of the temporal lobe was created as a subregion of the Mirrione cortex VOI (Supplemental Fig. S1). Standardized uptake values (SUV) for [^18^F]UCB-H and [^18^F]GE180 (60–90 min p.i.) as well as [^18^F]FDG (30–60 min p.i.) were calculated for the static late-phase time. In addition, volumes-of-distribution (V_T_) were computed for [^18^F]UCB-H image data on the basis of an image derived input function (IDIF), as has been described by Logan et al. (Logan et al., 1990). For this purpose, we obtained a blood input curve from a standardized VOI of the left ventricle (3 mm) with a maximum error tolerance of 10% and a threshold of 0. No corrections for radiometabolites were applied. However, according to a recent study conducted by Goutal et al. in 2021 (Goutal et al., 2021), the metabolite-corrected plasma input function as well as the plasma to whole-blood ratio of [^18^F]UCB-H SUV has been shown to be stable both within and between non-human primates. We therefore assumed a stable metabolization across all models and ages.

### 2.4 Immunohistochemistry and synaptic puncta analysis

Post-mortem mouse brain processing was performed as described elsewhere (Xiang, 2021, Briel et al., 2021). In brief, parasagittal slices were cut from paraformaldehyde-fixed hemispheres at 50 µm thickness using a VT1000S vibratome (Leica, Germany). From each animal 2–3 free-floating slices were stained immunofluorescently for SV2A. The protocol included permeabilization (2% Triton X-100 in PBS) and blocking (10% normal goat serum) steps before incubation with diluted primary antibodies (SV2A; 1:200, #ab32942; Abcam, Germany). After several washing steps secondary antibody goat anti-rabbit AlexaFlour®488 (1:1000, #A11008; Thermo Fisher Scientific, Germany) was applied. DAPI (1:1000, #D9542; Sigma-Aldrich, Germany) was used as nuclei counterstain. Slides were mounted on Superfrost-plus® slides (Thermo Fisher Scientific, Germany) with Fluorescence Mounting Medium (#S302380-2, Agilent Dako, Germany) and covered with #1.5H high-precision imaging glass coverslips (#48393-059, VWR, Germany).

Synapse imaging has also already been described in previous studies (Xiang, 2021, Briel et al., 2021). In brief, a Zeiss LSM780 confocal system (Zeiss, Germany) with Plan-Apochromat 40X (NA 1.4, DIC M27, oil immersion) objective was used. At constant settings, across 2–3 slices per animal 5–9 high-resolution images (image depth = 16-bit, dpi = 2048) of size (101.21 × 101.21) µm^2^ from the Somatosensory Cortex, Layers II/III, (75.87 × 75.87) µm^2^ from the hippocampal CA1, Stratum radiatum (PS2/APP cohort), the medial vestibular nucleus (VM) located in the brainstem and the nucleus interpositus of the cerebellum, and (48.87 × 75.87) µm^2^ from the hippocampal CA1, Stratum radiatum (P301S cohort) were sampled randomly. Image processing was automized with custom IJ2 macros in Fiji/ImageJ, including background subtraction, thresholding, and 'Analyze Particles' for puncta metrics.

### 2.5 Statistics

For each target VOI, a Shapiro-Wilk test was computed to test for normality of the data, followed by a Bartlett test to check for homogeneity of variances. When both tests proved insignificant across all target regions, multivariate group comparisons for each mouse model were carried out using a one-way ANOVA with post-hoc Tukey adjustment. If normal distribution and homogeneity of variances was not met, a Kruskal-Wallis Test followed by post-hoc pairwise Wilcoxon-Mann-Whitney-tests was computed. A p-value of 0.05 or less was considered significant. Furthermore, we explored the association between the [^18^F]UCB-H and [^18^F]FDG signal as well as [^18^F]FDG and [^18^F]GE180 uptake in a subset of mice by means of Pearson’s correlation coefficients and linear regression. All descriptive and inference statistics were computed using R Core Team (2022).

## 3 Results

### 3.1 [^18^F]UCB-H tracer kinetics indicate rapid influx and washout from brain parenchyma

**Fig. 1:**
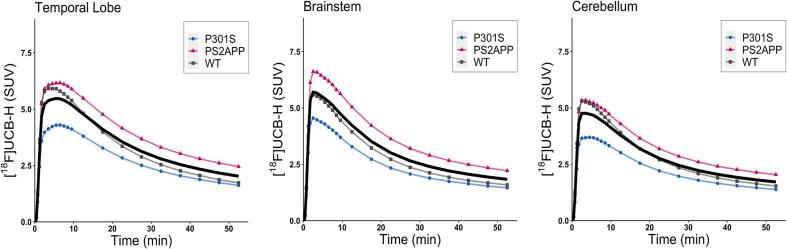
Time activity curves (TAC) of [^18^F]UCB-H (SUV) showed a similar influx peak after approximately five minutes followed by a continuous washout of the radiotracer in all three target regions. Mean TAC across all cohorts is represented by a black graph.

Averaged [^18^F]UCB-H time activity curves (0–60 min) of standardized uptake values (SUV) for each genotype and region of interest showed an influx peak after about five minutes post injection. This was followed by a continuous decline in radioactive signaling corresponding to a fast tracer washout from the regional brain tissue (Fig. 1).

### 3.2 Low variability of [^18^F]UCB-H uptake in the upper midbrain region suggests suitability as a pseudoreference region

**Fig. 2:**
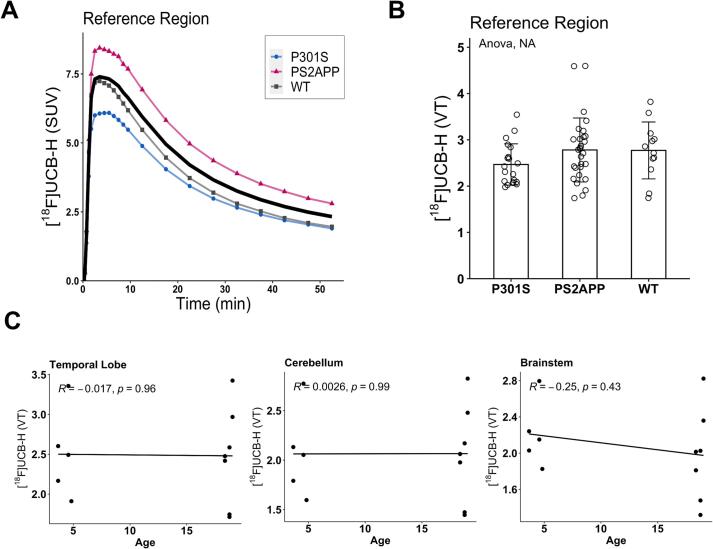
**(A)** Time activity curves of the reference region (upper midbrain) for each cohort showed equivalent kinetic features when compared to target regions. **(B)** Multivariate statistics (ANOVA) revealed no significant differences in [^18^F]UCB-H binding in the reference region across all three cohorts. **(C)** Scatter plots show no significant correlations between age and [^18^F]UCB-H binding (V_T_) in the wild-type cohort across all target regions.

First, we computed V_T_ images with IDIF to allow unbiased assessment of SV2A quantification across the brain. Exploratory analyses of [^18^F]UCB-H binding in VOIs predefined by the Mirrione atlas revealed least variation of uptake in an upper midbrain region, dominantly composed of white matter (CoV = 22.6%), across all investigated mouse models and wild-type mice. Pairwise comparisons of corresponding V_T_ values using Bonferroni-corrected t-tests showed no significant differences between PS2APP, P301S and wild-type mice for any age-related cohort (Fig. 2**B and** Supplemental Table S1). Additionally, an intra-cohort ANOVA of the PS2APP strain as well as t-tests of the respective P301S and wild-type cohorts also did not show any significant differences in the VT signal of this region between the different age groups (PS2APP: p = 0.11; P301S: p = 0.37; WT: p = 0.19). Consequently, we selected the upper midbrain region as a pseudoreference tissue for the computation of V_T_ ratios in further quantitative comparisons. Furthermore, no significant correlations were found between [^18^F]UCB-H uptake (V_T_) and age in the wild-type cohort (Fig. 2**C**). Therefore, all wild-type mice were pooled into a larger and more robust control sample.

### 3.3 Reduced [^18^F]UCB-H uptake portrays progressive synaptic loss in PS2APP and P301S mice

**Fig. 3:**
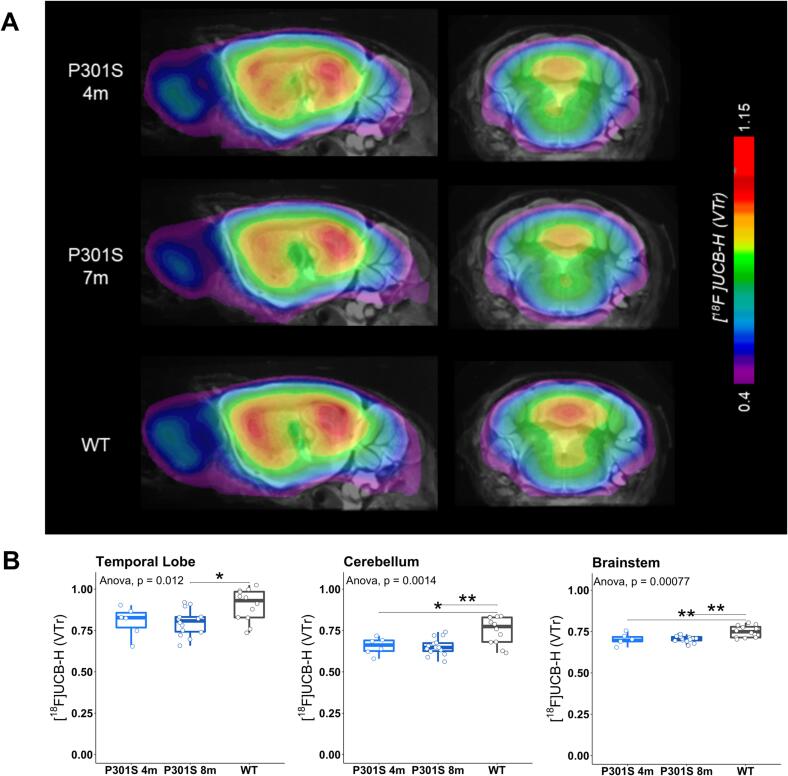
**(A)** Coronal sections of averaged [^18^F]UCB-H images (V_T_ ratios) show all target regions projected onto a standard MRI atlas for both groups of P301S transgenic mice and wild-type controls. **(B)** Boxplots of [^18^F]UCB-H binding (V_T_ ratios) across both groups of P301S mice showed significant SV2A reductions in all target regions when compared to wild-type controls. *p ≤ 0.05; **p ≤ 0.01; ***p ≤ 0.001.

To test whether there were statistically significant differences in [^18^F]UCB-H binding across the target regions of transgenic models, we next conducted multivariate analyses with post-hoc pairwise comparisons of V_T_ ratio values referenced to the upper midbrain region. By means of validation, we also performed regression analyses on the V_T_ ratio and distribution volume ratios (DVR) values of all target regions across all mice which revealed a very high correlation between the two parameters (R = 0.95, p < 2.2e-16; Supplemental Fig. S2). Both young and old P301S mice showed significantly lower tracer binding than wild-type mice in the cerebellum (4 m vs. WT: p = 0.018; 8 m vs. WT: p = 0.0018; Fig. 3) and the brainstem (4 m vs. WT: p = 0.0076; 8 m vs. WT: p = 0.0014; Fig. 3). Additionally, a significantly decreased [^18^F]UCB-H binding in the temporal lobe was found in 8-months old P301S mice when compared to wild-type controls (p = 0.014).

**Fig. 4:**
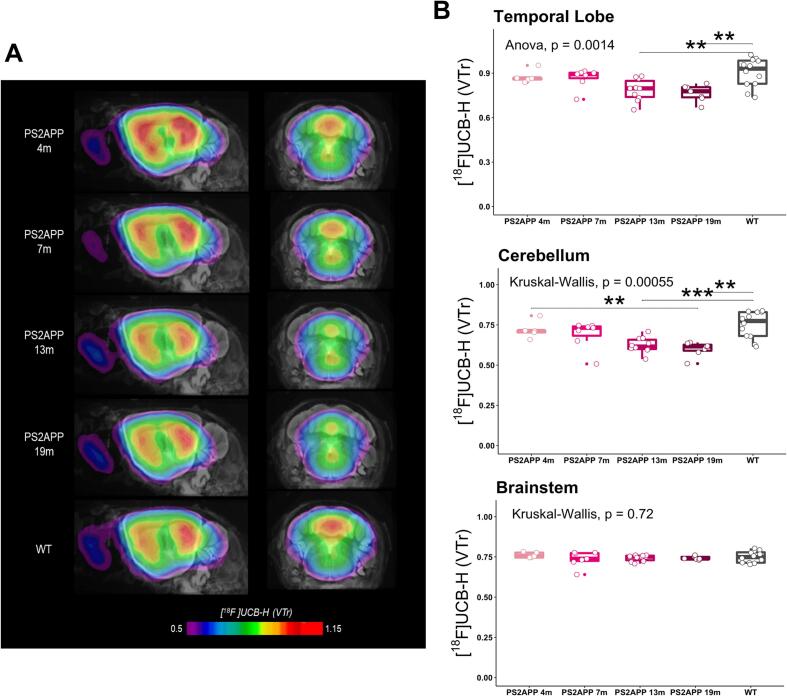
**(A)** Coronal sections of averaged [^18^F]UCB-H images (V_T_ ratios) show all target regions projected onto a standard MRI atlas for all age groups of PS2APP transgenic mice and wild-type controls. **(B)** Boxplots of [^18^F]UCB-H binding (V_T_ ratios) across all ages of PS2APP mice indicate significant SV2A reductions in the temporal and cerebellar target regions when compared to wild-type controls. *p ≤ 0.05; **p ≤ 0.01; ***p ≤ 0.001.

With regards to the PS2APP model, significantly reduced [^18^F]UCB-H binding was detected in the temporal lobe of both 13-months (p = 0.0080) and 19-months (p = 0.0042) old mice when compared to wild-type controls (Fig. 4). As the data did not allow for parametric comparisons, non-parametric Kruskal-Wallis tests were computed for the brainstem and cerebellum in PS2APP mice. Post-hoc pairwise Wilcoxon-tests revealed significantly lower [^18^F]UCB-H binding in 13-months (p = 0.006) and 19-months (p = 0.011) old PS2APP as opposed to wild-type mice in the cerebellum (Fig. 4). Brainstem V_T_ ratios did not show any significant differences between PS2APP and wild-type mice. With regards to the younger PS2APP cohorts (4 m, 7 m), no significant differences in V_T_ ratios were found in neither of the three target regions.

### 3.4 Synaptic loss is confirmed by immunohistochemical staining pattern in both PS2APP and P301S mice compared to wild-type controls

**Fig. 5:**
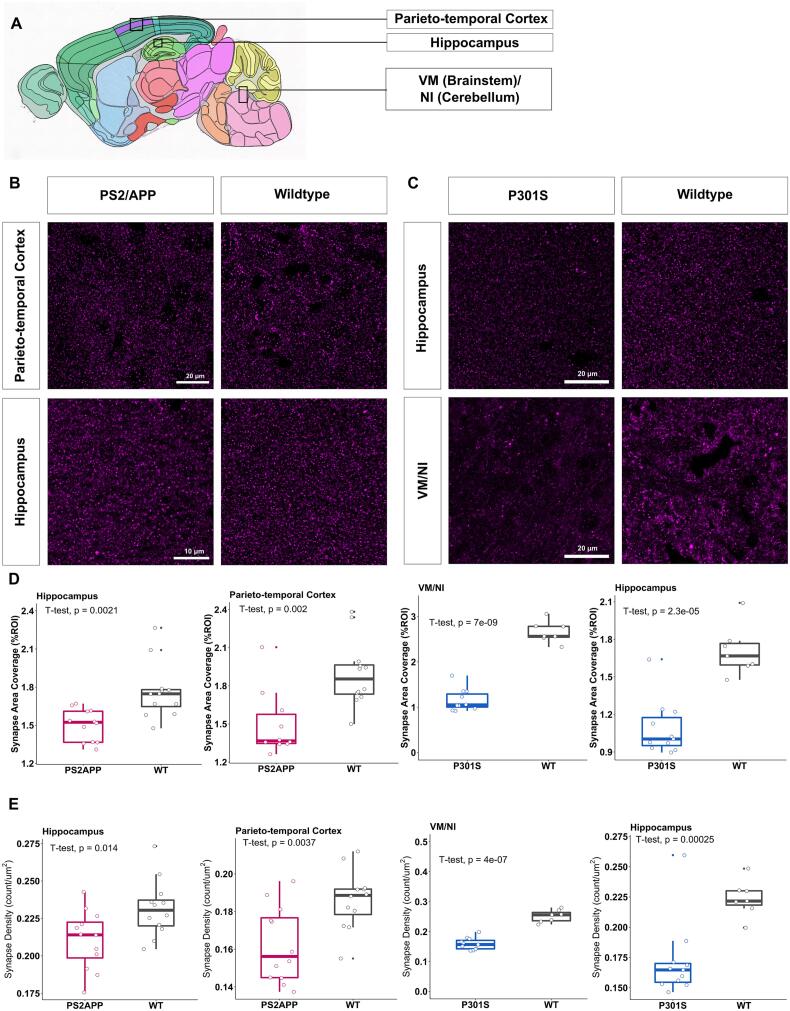
**(A)** Overview of sagittal mouse brain highlighting the three brain regions of interest parieto-temporal cortex, hippocampus, and ventral medial nucleus/ nucleus interpositus (VM/NI) from the Allen Mouse Brain Atlas (https://atlas.brain-map.org/atlas). **(B, C)** Immunofluorescence signal of SV2A compared across the parieto-temporal cortex and hippocampus in the PS2APP cohort and across the VM/NI and hippocampus in the P301S cohort. **(D, E)** Boxplots of SV2A-positive synapse coverage respectively SV2A-positive synapse in the cortex, hippocampus, and VM/NI of PS2APP/ P301S transgenic mice, which showed significant reduction of both indices in transgenic mice when compared to wild-type controls.

Immunohistochemical staining of SV2A was performed in the parieto-temporal cortex, the hippocampus, and the VM/NI as target regions of interest (Fig. 5**A, B**) (Institute, 2004) to evaluate the synaptic SV2A coverage and synaptic density in parallel to the PET results. A reduced synaptic SV2A coverage was observed in PS2APP mice when compared to wild-type controls in hippocampus (p = 0.0021) and parieto-temporal cortex (p = 0.002), and in P301S mice compared to wild-type controls in hippocampus (p = 2.3e-05) and VM/NI (p = 7e-09) (Fig. 5**D**). Similar results could be observed for synapse density of PS2APP mice when compared to wild-type controls in hippocampus (p = 0.014) and parieto-temporal cortex (p = 0.0037) and in P301S mice compared to wild-type controls in hippocampus (p = 0.00025) and VM/NI (p = 4e-07) (Fig. 5**E**).

### 3.5 Reduced [^18^F]UCB-H binding is associated with increased [^18^F]FDG uptake in PS2APP mice

**Fig. 6:**
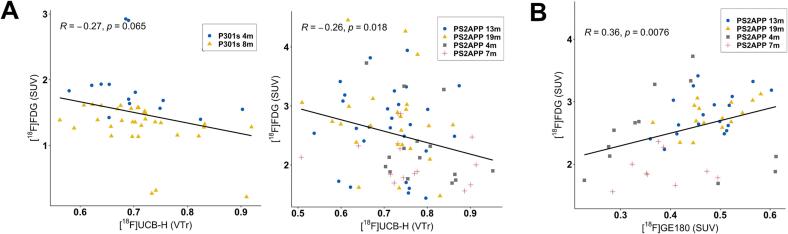
**(A)** Scatter plots show a significant negative correlation between [^18^F]UCB-H (V_T_ ratio) and [^18^F]FDG (SUV) signal across all target regions in PS2APP mice. **(B)** A significant positive correlation was found between [^18^F]FDG (SUV) and [^18^F]GE180 (SUV) in the PS2APP cohort.

To test whether synaptic loss as expressed by reduced [^18^F]UCB-H binding was associated with regional glucose metabolism, we calculated Pearson’s coefficient of correlation for [^18^F]UCB-H V_T_ ratio and [^18^F]FDG SUV across all target regions for both P301S and PS2APP genotypes (Table 2). As shown in Fig. 6**A**, no significant correlations between both biomarkers were found in the P301S model. However, there was a significant negative correlation between [^18^F]UCB-H binding and [^18^F]FDG uptake in the PS2APP cohort (R = − 0.26, p = 0.018), i.e. lower [^18^F]UCB-H binding corresponded to elevated glucose metabolism. To further investigate a probable reason for this inverse relationship, we also calculated Pearson’s correlation coefficient for [^18^F]FDG and [^18^F]GE180 SUV in the same PS2APP mice. Here, a positive relationship between the microglial signal and regional glucose metabolism was observed (Fig. 6**B**; R = 0.36, p = 0.008).

**Table 2:** Overview of glucose uptake in the cross-sectional preclinical study. Small-animal PET SUVs are shown for [^18^F]FDG.

Mouse Model	Age (mo)	[^18^F]FDG Small Animal Pet (n)	Cerebellum [^18^F]FDG signal (SUV_CB_)	Brainstem [^18^F]FDG signal (SUV_BS_)	Temporal Lobe [^18^F]FDG signal (SUV_TL_)
PS2APP	4.8	5	2.41 ± 0.76	2.38 ± 0.58	2.09 ± 0.67
	7.5	5	2.18 ± 0.44	2.26 ± 0.41	1.88 ± 0.37
	14.0	9	2.74 ± 0.73	2.75 ± 0.75	2.45 ± 0.65
	19.9	8	2.88 ± 0.80	2.80 ± 0.75	2.52 ± 0.68
P301S	4.8	4	1.83 ± 0.13	1.78 ± 0.11	1.48 ± 0.09
	7.4	10	1.49 ± 0.14	1.42 ± 0.14	1.26 ± 0.10

WT	4.7	6	1.60 ± 0.33	1.58 ± 0.27	1.43 ± 0.17

## 4 Discussion

The use of molecular imaging with PET in mouse models expressing specific disease-defining pathologies provides a unique opportunity to explore the precise interplay between different biomarkers. With regards to AD research, many radiotracers targeting amyloidosis and tau aggregates have already been well-established, contributing to a more detailed understanding of the temporal and spatial dynamics of the disease’s pathomechanisms (Villemagne et al., 2018). However, using [^18^F]FDG as a preclinical PET biomarker for neurodegeneration has so far yielded inconsistent data, thus driving the search for alternative molecular targets. In this study we investigated the capability to image progressive loss of synaptic density by means of [^18^F]UCB-H in parallel to [^18^F]FDG-related glucose metabolism in two distinct mouse models expressing major neuropathological characteristics of AD. This fluorine-18 labelled radiotracer selectively binds to the synaptic vesicle glycoprotein 2A and is therefore considered a surrogate biomarker for (pre-)synaptic density (Becker et al., 2020). Importantly, no on-site cyclotron is needed for [^18^F]UCB-H to be used practically, thus making it an excellent candidate for broad clinical use.

Because SV2A is ubiquitously expressed throughout the entire brain, defining a proper reference region for the purpose of normalizing [^18^F]UCB-H data is not entirely possible (Serrano et al., 2019). We therefore sought to instead define a pseudoreference region which would express the least variability in [^18^F]UCB-H uptake across all mouse models and target VOIs. Although the cerebellum is commonly used as a reference tissue in human studies of AD due to it being typically spared of amyloid debris (Chen et al., 2021), various preclinical observations have so far indicated considerable differences in cerebellar [^18^F]FDG signal between transgenic and wild-type mice (Deleye et al., 2016). Therefore, other brain regions have been proposed for preclinical [^18^F]UCB-H PET standardization, most commonly white matter regions with low specific binding characteristics such as the centrum semiovale (Nabulsi et al., 2016, Chen et al., 2018). However, reliably defining white matter regions in the mouse brain is quite challenging and hence prone to distortion. We therefore decided to base our reference tissue selection on statistical analyses of variation coefficients applied to predefined Mirrione atlas VOIs (Ma et al., 2005). To generate a robust area, we further combined upper midbrain VOIs with comparatively low variability and only few grey matter nuclei into one standardized reference VOI that we then used to quantify [^18^F]UCB-H uptake by means of V_T_ ratios.

In P301S transgenic mice with pathological tau aggregation, our findings revealed significant decreases of [^18^F]UCB-H uptake already at 4 months of age, which aggravated in 8-months old P301S mice when compared to wild-type controls. Decreases in synaptic density, as expressed by lower V_T_ ratio values, were found in all three target regions, i.e. the temporal lobe, brainstem and cerebellum at 8 months of age. These observations fit well with previous research causally linking neurotoxic tau aggregates to synaptic dysfunction and loss in AD models (Lasagna-Reeves, 2011) and primary tauopathies (Bigio et al., 2001, Briel et al., 2022). It is important to note, however, that this process presumably precedes the occurrence of the main pathological aggregate of tau, i.e. neurofibrillary tangles (NFTs), whose accumulation is strongly correlated with the onset and progression of clinical symptoms in humans (Arriagada et al., 1992, Long and Holtzman, 2019). In our study, this is particularly well illustrated in the P301S transgenic mouse model, which already shows decreased [^18^F]UCB-H signal by the age of 4 months in the cerebellum and brainstem. This finding is supported by previous studies observing synaptic impairment in 3-months old P301S mice long before the occurrence of NFTs at the age of 6 months (Yoshiyama et al., 2007). Taken together, this suggests that synaptic loss is a separate early entity of neurodegeneration which might facilitate preventive detection and timely intervention of AD-related pathomechanisms even before they manifest themselves clinically.

The second mouse model Included in this study represents another main feature of AD pathology, namely amyloidosis. Similar to our observations in P301S mice, we found significant reductions in [^18^F]UCB-H signal in the temporal lobe and cerebellum, but not the brainstem, in both 13- and 19-months old transgenic mice when compared to wild-type controls. This shows that synaptic loss can not only triggered by neurotoxic tauopathy but also in presence of Aβ. In line, previous studies concluded that the accumulation of oligomeric forms of Aβ contribute to a reduction in synaptic density and function as well by directly targeting synaptic spines (Lacor, 2007, Lacor, 2004). Similar to tau oligomers and NFTs, this synapto-toxic form of Aβ is a separate entity to the insoluble amyloid plaques, whose presence is not directly related to synaptic decline (Einstein et al., 1994). Furthermore, our preclinical observations fit well with human studies, which found significantly reduced [^18^F]UCB-H and [^11^C]UCB-J tracer uptake in the median temporal lobe and more specifically the hippocampus of AD patients in comparison to heathy controls (Bastin et al., 2020, Chen et al., 2018). This synapse loss correlates strongly with cognitive decline in humans and is again supposed to occur even before the onset of amyloidosis, thus providing valuable insights into early disease stages (Scheff et al., 2006).

As part of our multi-tracer approach, we further compared the regional glucose metabolism in all target regions to previously mentioned quantitative [^18^F]UCB-H uptake. We hypothesized that SV2A is a more specific biomarker of neuronal injury since glucose uptake in the brain is not restricted to neurons only but also driven by astroglial cells (Xiang, 2021, Zimmer et al., 2017). As a consequence, studies using [^18^F]FDG to measure hypometabolism observed that inflammatory glial cells infiltrating ischemic regions consume a disproportionate amount of glucose, thus distorting the actual extent of neurodegenerative decay (Backes et al., 2016). Contrary to previous findings (Endepols et al., 2022), we did not observe a significant positive association between glucose uptake and SV2A expression in either transgenic mice strain, but instead found that [^18^F]UCB-H and [^18^F]FDG signals were inversely associated with each other on a significant level in the PS2APP mouse strain while also showing a distinctly negative trajectory in the P301S strain. This finding might well be due to a signal distortion by glial cells colocalized to synaptic loss, which consume particularly high levels of glucose when in a functionally active state (Xiang, 2021). In line with our earlier findings (Brendel et al., 2016), we further found a significant correlation between [^18^F]FDG and [^18^F]GE180 in PS2APP mice, an observation which supports the stated hypothesis that increases of glucose uptake are linked to microglial activation. Further adding to this, a recently published clinical study also observed much more widespread synaptic loss than hypometabolism in extrastriatal areas of a cohort of patients with Huntington’s Disease, thus concluding that [^18^F]FDG is not as sensitive in detecting early changes in the disease’s pathophysiology (Delva et al., 2022). In future preclinical research, it will be critical to further illuminate how glucose metabolism and synaptic decline are temporally and spatially linked to neuronal loss in order to justify the use of one or the other as a representative biomarker for neurodegeneration.

Several aspects should be mentioned as limitations for the interpretation of our results. First, a previous study has shown that P301S mice show generalized brain atrophy at 8 months of age, while synapse impairment was already detected at an even younger age of 3 months (Yoshiyama et al., 2007). Presence of atrophy needs to be considered for the interpretation of the SV2A-PET results in the P301S cohort since signal loss can be attributed to two reasons: synapse loss in the context of specific neurodegeneration and/or due to a general reduction of brain parenchyma. In this regard, we have not been able to perform a MRI-based partial volume effect correction in this study, so that we cannot rule out a signal interference through spill-over effects in particularly small volumes of interest. Missing PVE correction was also the reason for which we omitted the hippocampus as a target region as signal distortion due to the small size was to be expected. Furthermore, we have not been able to correct the image-derived input functions used in kinetic modelling for radiometabolites. A transfer of radiometabolite data from previous study results of non-human primates, to which we have referred in the methods section, is not entirely permissible since the metabolic profile of radiotracers can show considerable interspecies discrepancies. Future studies need to address radiometabolite analysis in mice and lacking radiometabolite data in this species need to be considered as a current limitation of [^18^F]UCB-H. Finally, in the present study, we were unfortunately unable to perform a validation of the pseudoreference region at different ages by immunohistochemistry or immunoblotting. Thus, age-related reductions of SV2A in any pseudoreference tissue may limit the ability to detect small biological effects by relative SV2A-PET quantification in target tissues.

## 5 Conclusion

In-vivo PET imaging using [^18^F]UCB-H reliably depicts progressive synaptic loss in two mouse models expressing the major characteristics of neurodegenerative disorders, i.e. tauopathy and beta-amyloid pathology. Due to distorting metabolic inconsistencies that have previously been observed with the use of [^18^F]FDG, we therefore suggest that [^18^F]UCB-H could be a more robust biomarker for neurodegeneration in preclinical AD research. As the onset of synapse loss is thought to occur even before other major pathological biomarkers, integrating [^18^F]UCB-H into the diagnostic protocol could prospectively facilitate the detection and treatment monitoring in the earliest of disease stages of human patients.

Declaration of interest.

Johannes Levin reports speaker fees from Bayer Vital, Biogen and Roche, consulting fees from Axon Neuroscience and Biogen, author fees from Thieme medical publishers and W. Kohlhammer GmbH medical publishers. In addition, he reports compensation for serving as chief medical officer for MODAG GmbH, is beneficiary of the phantom share program of MODAG GmbH and is inventor in a patent “Pharmaceutical Composition and Methods of Use” (EP 22 159 408.8) filed by MODAG GmbH, all activities outside the submitted work.

## 6 Funding sources

This work was supported by Projekt DEAL through Open Access funding. This work was supported by grants from the Deutsche Forschungsgemeinschaft (DFG, German Research Foundation) under Germany’s Excellence Strategy within the framework of the Munich Cluster for Systems Neurology (EXC 2145 SyNergy – ID 390857198).

## Declaration of Competing Interest

The authors declare that they have no known competing financial interests or personal relationships that could have appeared to influence the work reported in this paper.

## Data Availability

Data will be made available on request.
