# Supplementary material for: Assessment of synaptic loss in mouse models of β-amyloid and tau pathology using [18F]UCB-H PET imaging
Source: Neuroimage Clin. 2023 Jul 26;39:103484. doi: 10.1016/j.nicl.2023.103484 (PMC10407951; doi:10.1016/j.nicl.2023.103484)
Supplement: Supplementary data 1 [file mmc1.docx]

**Supplemental Material to “Assessment of synaptic loss in mouse models of β-amyloid and tau pathology using [^18^F]UCB-H PET imaging”**

Letizia Vogler*^1^, Anna Ballweg*^1^, Bernd Bohr*^1^, Nils Briel^4^, Karin Wind^1^, Melissa Antons^1^, Lea Kunze^1^, Johannes Gnörich^1^, Simon Lindner^1^, Franz-Josef Gildehaus^1^, Karlheinz Baumann^5^, Peter Bartenstein^1^, Guido Boening^1^, Sibylle I. Ziegler^1^, Johannes Levin^2,3,6^, Andreas Zwergal^6,7^, Günter U. Höglinger^2,6^, Jochen Herms^4^, Matthias Brendel^1,2,3^

^1^Department of Nuclear Medicine, University Hospital of Munich, Ludwig-Maximilians-University (LMU) Munich, Munich, Germany
^2^German Center for Neurodegenerative Diseases (DZNE), Munich, Germany

^3^Munich Cluster for Systems Neurology (SyNergy), Munich, Germany

^4^Center for Neuropathology, LMU Munich, Munich, Germany
^5^ Roche Pharma Research and Early Development, Neuroscience Discovery, Roche Innovation Center Basel, F. Hoffmann-La Roche Ltd., Basel, Switzerland

^6^Department of Neurology, University Hospital of Munich, LMU Munich, Munich, Germany

^7^German Center for Vertigo and Balance Disorders (DSGZ), University Hospital of Munich, LMU Munich, Munich, Germany

**Supplemental Figures**


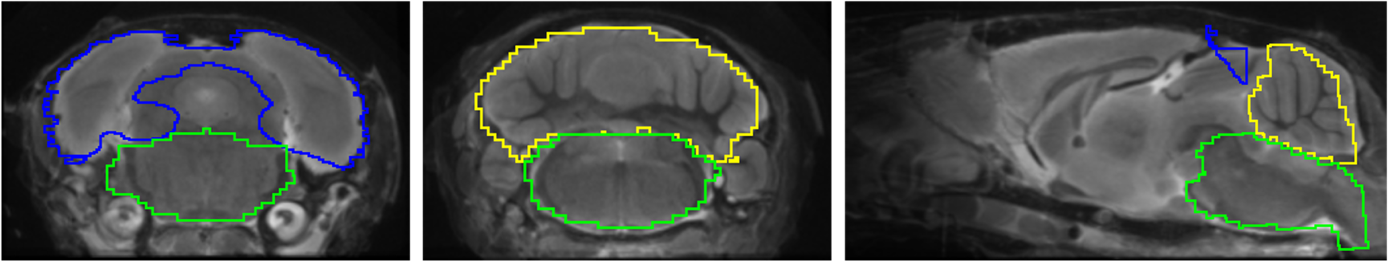

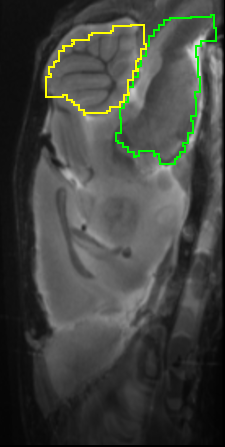


**Supplemental Figure 1**. Overview of the three target VOIs corresponding to the temporo-parietal cortex (blue), the cerebellum (yellow) and the brainstem (green).

**
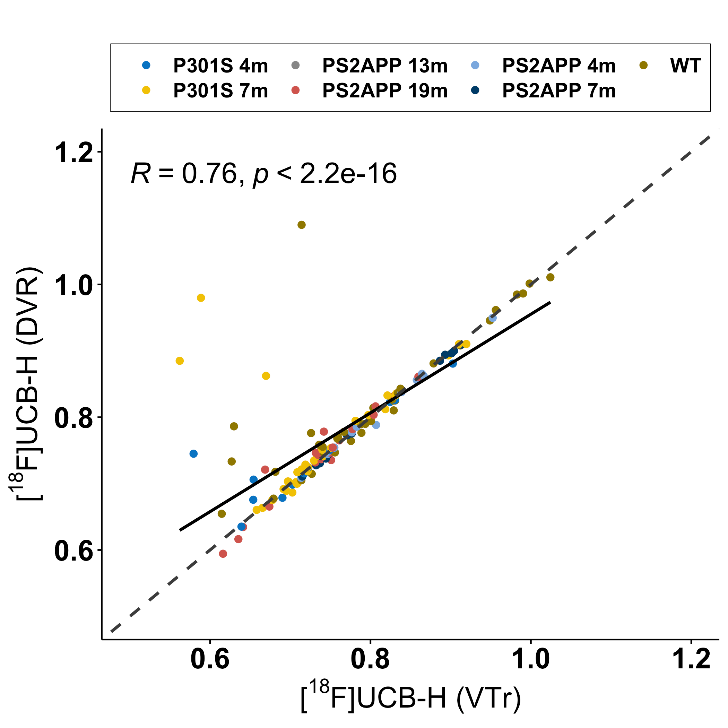
**

**Supplemental Figure 2.** Scatter plot of the V_T_ ratios and distribution volume ratios (DVR) across all target regions and mice models with a regression line (black) and line of identity (black dotted line) revealing significantly high correlations between the two parameters.

**Supplemental Tables**

| Mouse Model | Age (mo) | [^18^F]UCB-H Small Animal PET (n) | [^18^F]UCB-H Small Animal PET (V_T Ref_) |
| --- | --- | --- | --- |
| PS2APP | 4.4 | 5 | 2.97 ± 0.38 |
|  | 7.1 | 7 | 3.37 ± 0.87 |
|  | 13.5 | 10 | 2.57 ± 0.63 |
|  | 19.8 | 7 | 2.36 ± 0.24 |
| P301S | 4.3 | 6 | 2.63 ± 0.51 |
|  | 8.4 | 14 | 2.40 ± 0.41 |
| WT | 12.7 | 12 | 2.77 ± 0.61 |

**Supplemental Table 1.** Overview of the V_T_ values of the reference region (upper midbrain) across all mouse models and cohorts. Ref = Reference Region.
